# Platelet Turnover Predicts Outcome after Coronary Intervention

**DOI:** 10.1160/TH16-10-0785

**Published:** 2017-02-23

**Authors:** Matthias K. Freynhofer, Liana Iliev, Veronika Bruno, Miklos Rohla, Florian Egger, Thomas W. Weiss, Wolfgang Hübl, Martin Willheim, Johann Wojta, Kurt Huber

**Affiliations:** 1 3^rd^ Medical Department, Cardiology, Wilhelminenhospital, Vienna, Austria; 2 Ludwig Boltzmann Cluster for Cardiovascular Research, Vienna, Austria; 3 Department of Obstetrics and Gynecology, Wilhelminenhospital, Vienna, Austria; 4 Department of Laboratory Medicine, Wilhelminenhospital, Vienna, Austria; 5 Department of Cardiology, Medical University of Vienna, Vienna, Austria; 6 Medical Faculty, Sigmund Freud University, Vienna, Austria

**Keywords:** Reticulated platelets, mean platelet volume, Multiplate, VASP-P, dual antiplatelet therapy

## Abstract

Elevated platelet turnover contributes to high platelet reactivity. High platelet reactivity after percutaneous coronary intervention (PCI) is associated with major adverse cardiovascular events (MACE). The purpose of this study was to determine the prognostic value of platelet turnover and function with regard to MACE after PCI with stent implantation. In this prospective observational study, 486 consecutive patients after PCI on aspirin and clopidogrel were included to determine platelet turnover (mean platelet volume (MPV), reticulated platelet fraction (RPF)) and platelet function (multiple electrode aggregometry (MEA), vasodilator-stimulated phosphoprotein-phosphorylation (VASP-P) assay). At six-months follow-up, MACE occurred in 10.7 % of patients. RPF (odds ratio [OR]=1.173 (95% confidence interval [CI 95 %] 1.040–1.324), p=0.009) and MPV (OR=1.459 (CI 95 % 1.059–2.008), p=0.021) were univariable predictors of MACE, whereas VASP-P (OR=1.016 (CI 95 % 1.000–1.032), p=0.052) and MEA (OR=0.999 (CI 95 % 0.980–1.017), p=0.895) failed to predict MACE. RPF remained the only platelet variable independently associated with MACE. The best model to predict MACE included: troponin I (OR=1.007 (CI 95 % 1.002–1.012), p=0.009), RPF (OR=1.136 (CI 95 % 1.001–1.288), p=0.048), CRP (OR=1.008 (CI 95 % 1.001–1.014), p=0.023) and history of myocardial infarction (OR=2.039 (CI 95 % 1.093–3.806), p=0.025). RPF (OR=1.211 (CI 95 % 1.042–1.406), p=0.012) was also independently associated with in-hospital bleedings. In conclusion, RPF as index of platelet turnover is an independent predictor of MACE and bleeding events in PCI patients on dual antiplatelet therapy. Since RPF can reliably be quantified along with routine haemograms, RPF might easily be applied in the setting of cardiovascular risk prediction.

## Introduction

Platelets are a pivotal element in primary haemostasis and repair of endothelial damage. Yet, platelets are also essentially involved in initiation and propagation of atherosclerosis and acute coronary syndromes (1). Acetylsalicylic acid (ASA) and clopidogrel are beneficial in patients with acute coronary syndromes or after percutaneous coronary intervention (PCI) (2–4). However, low-response to clopidogrel, i. e. high on- (clopidogrel-) treatment platelet reactivity (HTPR), has been linked to adverse ischaemic outcome in numerous investigations and *vice versa*, good response to clopidogrel might be associated with increased risk of bleeding (5). Furthermore, several routinely available (physical) platelet variables might also help to identify patients with hyperreactive platelets. Larger platelets are younger and more reactive compared to their counterparts (6–8). In situations with enhanced platelet turnover with release of new platelets from the bone marrow, the fraction of larger, and more reactive platelets also increases. Since mean platelet volume (MPV) is easy to measure it has widely been used as surrogate parameter of platelet turnover. There is evidence that MPV predicts myocardial infarction, death after myocardial infarction or restenosis following PCI (9), and the megakaryocyte-platelet system might even be causally involved in the initiation or propagation of atherosclerosis and acute coronary syndromes (ACS) (10). Yet, the principle to determine platelet turnover from peripheral blood affords a specific marker that identifies old versus new platelets. Decades ago, it was described that newly released platelets contain residual mRNA and rough endoplamatic reticulum, which could be stained (11). In analogy to reticulocytes, young platelets were termed reticulated platelets. Reticulated platelets are elevated in smokers and diabetics (12), in patients with stable coronary artery disease (CAD) (6, 13), suggesting an increased interaction of platelets with the atherosclerotic vessel wall (14), and in ACS patients (15). Reticulated platelets are more reactive (7) compared to older platelets and participate more eager in thrombus formation (16). The insufficient response to antiplatelet therapy might be explained either by their preserved ability to synthesize proteins of the alpha granules and of the final common pathway of platelet aggregation or by platelet turnover per se as new, non-inhibited platelets are released into the blood stream (7). However, outcome data in regard to platelet turnover are scarce (17).

We have therefore investigated the prognostic value of MPV and reticulated platelet fraction (RPF) as well as platelet function tests (multiple electrode aggregometry (MEA) and vasodilator-stimulated phosphoprotein-phosphorylation (VASP-P) assay), in regard to major adverse cardiovascular events (MACE) following PCI and stenting.

## Materials and methods

### Patients

**Figure 1:**
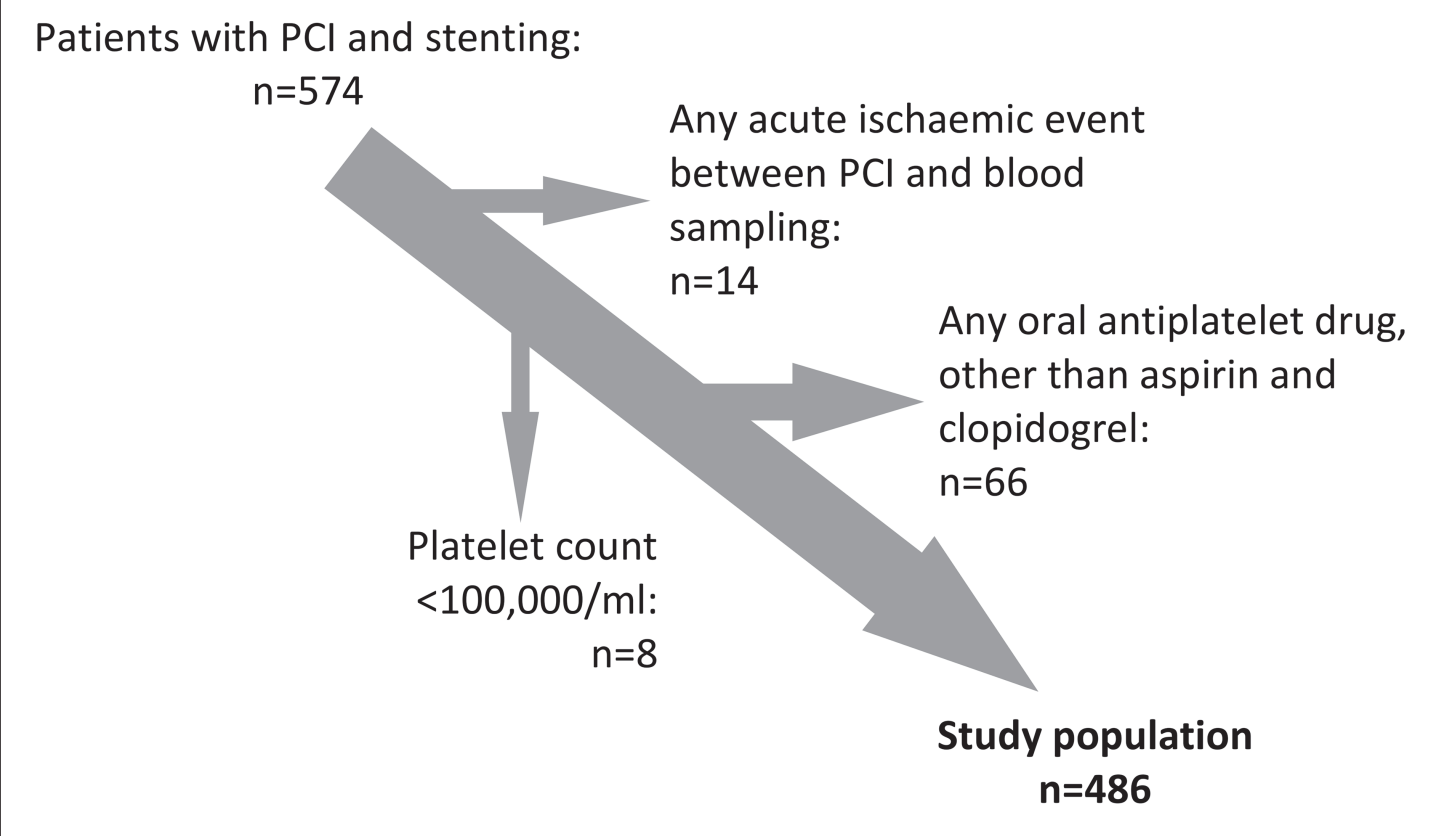
Patient flow diagram.

The Wilhelminenhospital Monitoring of Antiplatelet Activity (WILMAA)-registry is a prospective single center observational study. It includes 486 consecutive patients of the 3^rd^ Medical Department, Cardiology, Wilhelminenhospital, Vienna between May 2009 and December 2010. Patients who underwent PCI and coronary stenting with dual antiplatelet therapy aged >18 years were eligible. Among 574 recruited patients, 88 had to be excluded (► Figure 1). All participants gave their informed consent and the study was approved by the Ethics Committee of the City of Vienna.

### PCI, antiplatelet therapy and clinical management

Periprocedural clopidogrel was administered as previously described (18). Maintenance dose (MD) comprised aspirin 100 mg daily dose in combination with clopidogrel 75 mg daily dose administered in the mornings. The use of glycoprotein (GP)-IIb/IIIa-blockers during PCI as well as the choice of the anticoagulant depended on the individual situation and the thrombus load at angiography and was left to the discretion of the interventional cardiologist.

PCI procedures were carried out according to current guidelines. Stent type selection (bare metal stent or drug eluting stent) was left to the discretion of the operator.

### Data management and follow-up

Data were collected prospectively and entered into a central database. Follow-up information was obtained by outpatient visits and / or telephone interview. Source data of all possible events were collected.

Following the recommendation of the Academic Research Consortium regarding stent trials (19), the primary endpoint of WILMAA was defined as a composite of postprocedural major adverse cardiovascular events (MACEs), including: 1) cardiovascular death according to the TIMI-definition (www.timi.org), as well as any death that could not be attributed to non-cardiovascular reasons, 2) nonfatal myocardial infarction according to the TIMI-definition (www.timi.org), as well as 3) any unplanned revascularisation.

Additionally, 4) definite and probable stent thrombosis (ST) according to the academic research consortium definition (19), 5) unstable angina defined as ischaemic symptoms without elevation of troponin I above the upper limit of normal, and 6) transient ischaemic attack (TIA) (20) or stroke, defined as cerebral infarction were recorded. All events were classified by two physicians unaware of the platelet variables or platelet function test results.

Furthermore, the secondary endpoint comprised postprocedural bleedings during the index hospital stay, defined according to the TIMI haemorrhage classification scheme (www.timi.org). Any clinical overt sign of bleeding with a drop in haemoglobin levels ≥5.0g/dl or intracranial bleeding was defined as major whereas any clinical overt sign of bleeding with a drop in haemoglobin levels ≥3 was defined as minor bleeding.

### Blood sampling, Platelet characteristics

At 6-24 hours (h) after PCI (i. e. the next morning after PCI, after intake of the MD) venous blood samples along with routine blood samples were collected via venipuncture of the forearm into ethylenediaminetetraacetic acid tubes (Greiner BioOne, Kremsmünster, Austria) for the determination of platelet characteristics, coagulation tubes (buffered sodium citrate 3.2 %, Greiner BioOne), filled to maximum capacity for the VASP-P assay and into lithium-heparin tubes (Greiner BioOne) for MEA. The first tube was not used for platelet function testing.

Platelet count, MPV and reticulated platelets were measured by means of a Sysmex XE-2100 Automated Hematology System (Sysmex, Kobe, Japan (21)). The platelet count was determined both through impedance technology and an optical method, following interaction with a fluorescent dye which is found in the reticulocyte channel. MPV was calculated as: MPV (fl) = [(platelet (%) / platelet count (x109/l)]. Platelet RNA was stained with two fluorescent dyes, i. e. polymethine and oxazine. The cells were passed through a semiconductor diode laser beam and the forward scatter light (cell volume) and fluorescence intensity (RNA content) were determined. A computer algorithm (Sysmex IPF Master) discriminated the older and younger platelets by the intensity of forward scattered light and fluorescence. Reticulated platelets are displayed as percentage of the total optical platelet count. An approximate reference range from 1–5 % is indicated by Sysmex.

The VASP-P analysis was performed using PLT VASP/P2Y_12_ kits (Biocytex, Marseilles, France) according to the manufacturer’s instructions and as previously described (18). The platelet reactivity index (PRI, %) was calculated from the median fluorescence intensity.

The MEA (Multiple Platelet Function Analyser, Verum Diagnostica GmbH, Munich, Germany) high sensitive adenosine diphosphate (ADP)-assay was performed as previously described (18).

### Statistical analysis

Power and sample size considerations: Since the WILMAA-registry is the first investigation relating reticulated platelet fraction with MACE following coronary intervention with stent implantation, no meaningful sample size calculation was possible. The study is observational and exploratory in nature. It was initially planned to include 500 patients. Of 574 recruited patients, 88 patients had to be excluded and finally 486 patients were analysed.

Data are presented as mean ± SD for normally distributed continuous variables (normal distribution confirmed via Kolmogorov-Smirnov test and visual judgement of distribution curves) unless depicted otherwise. Median values (25^th^-75^th^ percentiles) are shown for skewly distributed continuous variables. Continuous variables were compared by t-test (paired or unpaired) or the Wilcoxon rank sum test (or the Wilcoxon signed rank test), as required. Categorical variables were compared by the Pearson’s Chi-square test or the Fisher’s exact test, as required.

Univariable logistic regression with regard to MACE at six months follow-up (and in-hospital bleeding) was performed including MEA, VASP-P, RPF, and MPV as well as age, gender, body mass index, bodyfat, indication for PCI, cardiogenic shock, traditional cardiovascular risk factors, history of PCI, history of myocardial infarction, history of atrial fibrillation, history of TIA/stroke, moderate and heavy alcohol consumption, heart rate at admission, systolic blood pressure at admission, blood glucose at admission, renal function (GFR), troponin I, platelet count, decline in platelet count, haemoglobin levels, leukocyte count, C-reactive protein, total cholesterlol, LDL-cholesterol, HDL-cholesterol, use of GPIIb/IIIa-blockers, type of anticoagulants during PCI, comedication, vascular access site, multivessel PCI, number of stents used, use of drug eluting stents, total stent-length and minor or major bleeding.

#### Multivariable logistic regression (MACE)

The independent association of the platelet parameters was tested in an automated multivariable logistic (forward conditional) regression model including potentially confounding variables or variables previously recognised to be associated with MACE (age, gender, indication for PCI, cardiogenic shock, traditional cardiovascular risk factors, heart rate at admission, systolic blood pressure at admission, renal function and troponin I) as well as all univariable predictors of MACE with p<0.05. Hosmer and Lemeshow test was applied to test for adequacy of the model.

#### Multivariable logistic regression (bleeding)

The independent association of platelet parameters with postprocedural bleeding risk during index hospital stay was tested in an automated multivariable logistic (forward conditional) regression model including potentially confounding variables or variables previously recognized to be associated with bleeding risk (age, gender, indication for PCI, cardiogenic shock, diabetes, history of TIA/stroke, heart rate at admission, systolic blood pressure at admission, haemoglobin, renal function and troponin I) as well as all univariable predictors of bleeding with p<0.05. Hosmer and Lemeshow test was then applied to test for adequacy of the model.

A receiver operating characteristic (ROC) curve analysis was calculated to determine the ability of RPF to distinguish between patients with or without MACE. The optimal cut-off was defined as the greatest sum of sensitivity and specificity. The predictive significance of RPF, as a dichotomised variable according to the calculated cut-off, for time to first MACE was furthermore tested in Kaplan–Meier analysis with log-rank test.

Determinants of RPF were explored by means of thoughtful variable selection, followed by automated (smallest akaike information criterion) linear regression modelling.

A two-sided p<0.05 was defined as significant. Data were processed using SPSS 20.0 (SPSS Inc., Chicago, Illinois), and Graph Pad Prism 5.0 (GraphPad Software Inc., La Jolla, CA, USA).

All authors had significant input into the study, had full access to and take full responsibility for the accuracy of the data and have read and agreed to the present manuscript.

## Results

### Patients

A total of 486 consecutive patients were enrolled. Mean age was 64.4±12.8 years and 31.7 % were women. The baseline characteristics are summarised in ► Table 1 and ► Table 2.

### Clinical endpoints

Six months follow-up was completed by 95.5 %. Two patients (0.4 %) were lost to follow-up and 20 (4.1 %) patients died during follow-up. Median follow-up duration was 190 (180–243) days. Overall, 86 ischaemic events, concluding 18 cardiovascular deaths, 21 nonfatal myocardial infarctions, 27 unplanned revascularisations, 4 definite stent thromboses, 6 probable stent thromboses, 6 unstable anginas and 4 TIA/Strokes occurred in 52 patients during follow-up. Mean time to first MACE was 104 ± 81 days.

Furthermore in 39 patients bleeding events of clinical significance, comprising 29 minor and 11 major bleedings, were recorded during index hospital stay. Mean time to first minor or major bleeding was 4.8±3.6 days.

### Prediction of MACE

In univariable logistic regression analysis, RPF (odds ratio [OR]=1.173 (95% confidence interval [CI 95 %] 1.040–1.324), p=0.009) and MPV (OR=1.459 (CI 95 % 1.059–2.008), p=0.021) were predictors of MACE, whereas VASP-P borderline (OR=1.016 (CI 95 % 1.000–1.032), p=0.052) and MEA clearly (OR=0.999 (CI 95 % 0.980–1.017), p=0.895) failed to significantly predict MACE (► Table 3).

In multivariable logistic regression, MACE was best predicted by troponin I (OR=1.007 (CI 95 % 1.002–1.012), p=0.009), RPF (OR=1.136 (CI 95 % 1.001–1.288), p=0.048), CRP (OR=1.008 (CI 95 % 1.001–1.014), p=0.023) and history of myocardial infarction (OR=2.039 (95 % CI 1.093–3.806), p=0.025).

**Figure 2:**
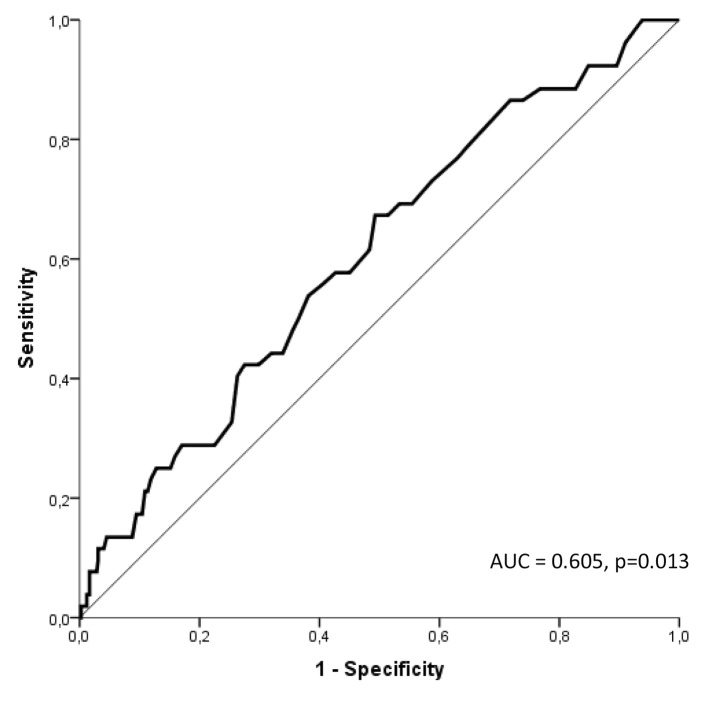
**ROC curve.** RPF distinguished significantly between patients with and without MACE at 6 months follow-up.

**Figure 3:**
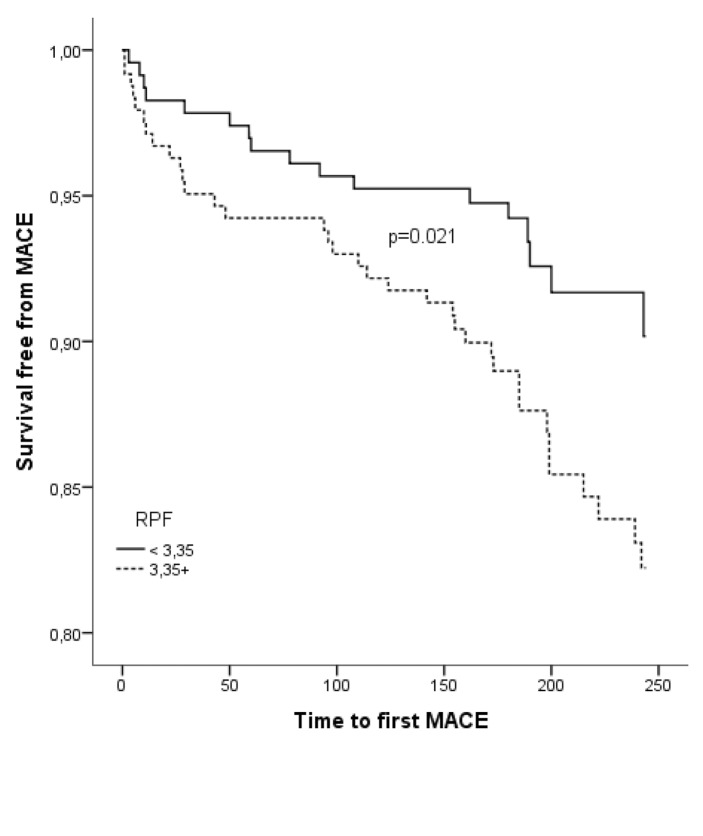
**Kaplan-Meier analysis.** The cumulative risk for MACE was significantly increased above the cut-off value of RPF ≥ 3.35 %.

According to ROC-curve analysis, RPF distinguished significantly between patients with and without MACE at six months follow-up (► Figure 2). The area under the ROC-curve was 0.605 (CI 95 % 0.525–0.685), p=0.013. The optimal cut-off-value was at RPF=3.35 % providing a sensitivity of 67 % and a specificity of 51 % for MACE. The positive predictive value was 14 %, whereas the negative predictive value exhibited 93 %. The MACE rate of 14.4 % in patients with RPF≥3.35 % was significantly higher compared to 7.4 % in patients with RPF<3.35. (p=0.014). The relative risk reduction was 48.2 % in the group of patients with low RPF compared to the group of patients with RPF≥3.35 %. Furthermore, in Kaplan-Meier analysis patients with RPF≥3.35 % had a significantly increased risk for MACE at six months follow-up (p=0.021), (► Figure 3). The single ischaemic endpoints according to the cut-off are listed in ► Table 4.

### Prediction of postprocedural bleeding (during index hospitalisation)

In univariable logistic regression analysis, RPF (OR=1.243 (95 % CI 1.091–1.415), p=0.001) and MPV (OR=1.484 (CI 95 % 1.035–2.127), p=0.032) were predictors of the composite of minor and major bleedings, whereas VASP-P (OR=1.015 (CI 95 % 0.997–1.034), p=0.099) and MEA (OR=0.991 (CI 95 % 0.970–1.013), p=0.418) failed to predict bleeding complications (► Table 3).

In multivariable logistic regression, bleeding was best predicted by: haemoglobin (OR=0.683 (CI 95 % 0.544–0.857), p=0.001, CRP (OR=1.013 (CI 95 % 1.005–1.021), p=0.001), GPIIb/IIIa-blocker use (OR=1.868 (CI 95 % 1.329–2.627), p<0.001), cardiogenic shock (OR=8.599 (CI 95 % 1.717–43.072), p=0.009) and RPF (OR=1.211 (CI 95 % 1.042–1.406), p=0.012).

### Determinants of platelet turnover

According to automated linear regression modelling, RPF (F=15,196; adjusted R-squared: 0.193; p<0.001) was inversely associated with platelet count (p<0.001), high density lipoprotein-levels (p=0.041), heart rate at admission (p=0.226) and body mass index (p=0.125), while it was positively predicted by diabetes with insulin therapy (p=0.006), leucocyte count (p=0.026), cardiogenic shock (p=0.004) and diabetes with oral antidiabetics (p=0.067).

### Platelet turnover at admission and changes over time

RPF was available in 329 (67.7 %) patients at the time of admission. The predictive value with regard to bleeding and ischaemic events was similar in this reduced sample (data not shown). MPV at the time of admission was available in 470 (96.7 %) patients and was neither associated with bleeding, nor with MACE (data not shown).

RPF at admission was comparable in patients suffering from stable coronary artery disease (CAD) (3.6 % (2.5–4.4)), Non ST-elevation-acute coronary syndrome (NSTE-ACS) (3.5 % (2.7–5.3)) and ST-elevation-acute coronary syndrome (STE-ACS) (3.7 % (2.8–5.2), p=0.211). We observed a significant decline from admission to postprocedural RPF in patients with STE-ACS (3.7 % (2.8–5.2) vs 3.6 % (2.7–4.8), p<0.001) and NSTE-ACS (3.5 % (2.7–5.3) vs 3.3 % (2.4–4.8), p<0.001), whereas there was no significant change in RPF following PCI in patients with stable CAD (3.6 % (2.5–4.4) vs 3.3 %(2.4–4.6), p=0.210).

## Discussion

The most important finding of the present investigation is that RPF as index of platelet turnover is an independent predictor of MACE in patients undergoing PCI with stenting on dual antiplatelet therapy with aspirin and clopidogrel. In contrast, MPV, VASP-P and MEA failed to independently predict MACE.

The risk of MACE in patients with RPF ≥3.35 % was about double that of patients below the cut-off. Cesari et al. recently investigated the association of RPF with cardiovascular death in a cohort of 229 ACS patients. Similarly to our investigation, an optimal cut-off at RPF=3.3 % was identified (22).

Thrombopoietin is the primary regulator of thrombopoiesis. It induces proliferation and differentiation of megakaryocyte progenitor cells as well as megakaryocyte maturation resulting in increased megakaryocyte size, polyploidisation and finally increased platelet production (1, 23). Early studies have found that increased megakaryocyte ploidy is present in patients with atherosclerosis, myocardial infarction as well as in patients with sudden cardiac death and that it is inversely correlated with bleeding time (24–26). Increased platelet production might be best represented in the fraction of younger platelets, containing megakaryocyte-derived mRNA. However, reticulated platelets are not only younger, but also larger (6), suggesting an overlap between the proportion of young platelets (reticulated platelets) and the proportion of larger platelets (MPV). There is evidence that MPV predicts myocardial infarction, death after myocardial infarction or restenosis following PCI (9) and the megakaryocyte-platelet system might even be causally involved in the initiation or propagation of atherosclerosis and ACS (10). Larger platelets are metabolically more active and hold a greater thrombogenic potential, comprising stronger platelet aggregation, increased synthesis of thromboxane as well as expression of more surface receptors, like GP Ib or IIb/IIIa (27). According to our data, MPV was a univariable predictor of MACE, but failed to independently predict outcome. Why RPF, but not MPV was independently predictive for MACE can only be hypothesised: MPV is only a measure of platelet size and not all large platelets are reticulated platelets. We therefore suggest that thrombopoiesis is more specifically reflected by measuring (functional) messenger RNA rather than platelet size alone. Furthermore MPV was also not independently associated with bleeding complications.

Although aspirin irreversibly inhibits platelet cyclooxygenase-1, recovery of platelet function occurs within a normal 24 h dosing interval in patients as well as in healthy controls treated with once-daily aspirin (28–31). Recent investigations have also described HTPR during aspirin treatment in association with elevated levels of reticulated platelets (7).

Explanations might include that the newly released platelets contain mRNA with the preserved abillity to produce glycoprotein IIb/IIIa receptors, COX-2 and procoagulant proteins of the alpha-granules including fibrinogen and von Willebrand factor (32–34). COX-2 might be especially relevant, since it is not sufficiently inhibited by low-dose aspirin, giving rise to augmented synthesis of TXA2. Furthermore, elevated platelet turnover itself might be important, since non-inhibited platelets are released into the blood stream and, depending on the turnover rate, might cause insufficient platelet inhibition, especially at the end of the 24-h dosing interval. Whether platelet turnover itself also contributes to impaired antiplatelet response to thienopyridines was recently questioned (35). However, even despite modern antiplatelet therapies (including prasugrel and ticagrelor), reticulated platelets aggregate stronger compared to normal platelets (36), and it has been hypothesised that increased platelet turnover might even result in severe complications like ST (7, 37). In contrast, our prospective data did not confirm these findings. However, this might simply be explained by the low rate of stent thromboses.

Outcome data in regard to reticulated platelet count or fraction are scarce (17). In 2012, Cesari et al. have shown in 229 ACS-patients that RPF independently predicts cardiovascular death (22). Others suggested from a smaller cohort that immture platelet count might represent a novel biomarker for MACE risk stratification. Ibrahim et al. have tested the predictive value of immature platelet count in 89 coronary artery disease patients treated with prasugrel in regard to a composite of all-cause mortality, myocardial infarction, unplanned revascularisation and hospitalisation for angina (38). The present investigation confirms that RPF independently predicts MACE and expands previous findings (22, 38), i. e. RPF independently predicts MACE in patients undergoing PCI with stenting.

On the other hand, it was surprising to find an independent association of increased postprocedural platelet turnover with bleeding complications. However, since platelet turnover (in a reduced sample) at admission was similarly associated with postprocedural haemorrhage it is plausible that platelet turnover precedes bleeding complications. We are not aware of any previous reports investigating platelet turnover and the risk of postprocedural bleedings. The conventional model, that reticulated platelets only indicate a pro-thrombotic situation, might therefore be re-considered. It remains highly speculative, but increased platelet turnover might rather be a marker of endothelial dysfunction/endothelial damage and severe vascular disease with the greater need to repair endothelial lesions than a pro-thrombotic marker alone. A severely diseased endothelium on the one hand might give rise to more ischaemic complications, but on the other hand might be dysfunctional to control bleedings. According to our data, RPF was independently predicted by several markers that might indicate endothelial dysfunction (i. e. low levels of high density lipoprotein, body mass index, diabetes and cardiogenic shock. Anyhow, these findings warrant further investigation.

Despite the beneficial effects of ASA and clopidogrel in patients with ACS and/or after PCI, there is broad evidence that HTPR increases the risk of ischaemic events, first and foremost, stent thromboses (5). In times of highly efficacious adenosine-diphosphate (ADP)-receptor blockers like prasugrel or ticagrelor balancing bleeding risks becomes increasingly important (5). We have previously reported univariable predictive value of VASP-P assay in the first 300 patients of the WILMAA registry with regard to definite and probable stentthromboses, STE-ACS and cardiovascular death (18). In the present investigation, VASP-P marginally (p=0.052) failed to predict MACE. Furthermore, VASP-P was not associated with a composite of TIMI minor or major bleeding during index hospital stay. MEA was neither associated with MACE nor with the composite of TIMI minor or major bleeding. For the WILMAA-registry the ADP high sensitivity assay was applied. It is of great importance to note that lithium-heparin tubes instead of the widely applied hirudin-tubes for blood collection were used. This indeed might explain the neutral findings in regard to MEA.

**What is known about this topic?**

- An elevated reticulated platelet fraction - as index of elevated platelet turnover - contributes to high platelet reactivity.
- High platelet reactivity after percutaneous coronary intervention is associated with major adverse cardiovascular events.

**What does this paper add?**

- An elevated platelet turnover independently predicts major adverse cardiovascular events after percutaneous coronary intervention.
- The reticulated platelet fraction - as index of elevated platelet turnover - might be superior in predicting ischaemic risk compared to mean platelet volume or specific platelet function tests.

Recent investigations have shown correlation of reticulated platelets with CAD, especially with ACS (15). This association could not be confirmed with our data. However, we could document a significant decline in platelet turnover following successful PCI with stenting in patients suffering from ACS.

Grove et al. have investigated determinants for increased platelet turnover in a cohort of patients with stable CAD under aspirin therapy. Patients with diabetes, active smokers, i. e. patients with endothelial dysfunction (1), display an increased platelet production with a higher fraction of more active circulating platelets (12). In our patients with stable CAD as well as with ACS under dual antiplatelet therapy, diabetes, leucocyte count and cardiogenic shock were significantly associated with increased platelet turnover. On the other hand, higher HDL-levels and a higher platelet count determined lower platelet turnover. As stated above, thrombopoietin is the primary regulator of thrombopoiesis and its plasma concentration varies inversely with platelet count (23), which explains the inverse association of platelet count with turnover. This association might furthermore explain – among other explanations (39) – the increased ischaemic risk of patients with postprocedural thrombocytopenia. The remaining determinants of platelet turnover represent markers of endothelial dysfunction and propagation of atherosclerosis (1).

### Limitations and strengths

One important limitation of our study is that we only included patients on dual antiplatelet therapy comprising aspirin and clopidogrel. Extrapolation of our results to newer ADP-receptor blockers might therefore be critical, although, a recent investigation has also described a correlation of increased platelet turnover with HTPR under prasugrel therapy (40). Furthermore we did not measure thrombopoietin, but the association of thrombopoietin with platelet turnover has previously been established and was beyond the scope of our investigation.

The prospective, but observational nature carries all limitations inherent in such an investigation. While great effort was made to adjust for differences in baseline variables, the potential for unrecognised confounders is always present in observational studies. Furthermore, as the study was exploratory in nature/hypothesis generating it was not powered to exactly determine the additional effect of platelet turnover to conventional/established risk scores. The study size does not allow to draw firm conclusions whether platelet turnover or platelet function are better markers for risk estimation in PCI patients. Further clinical investigations are therefore mandatory.

As the enrollment was strictly consecutive, a selection bias is unlikely and the investigated patients might be considered representative for an unselected, “real-life” CAD patient cohort.

### Conclusion

RPF as index of platelet turnover is an independent predictor of MACE but also of postprocedural bleeding events in patients undergoing PCI with stent implantation on dual antiplatelet therapy with aspirin and clopidogrel. We therefore speculate that increased platelet turnover might represent a marker of endothelial dysfunction/endothelial damage than a pro-thrombotic marker alone. Given the direct comparison of the predictive value of MPV and RPF, RPF might be superior to predict ischaemic risk. MPV has been extensively investigated since it is a cheap, fast and widely available platelet biomarker. However, in light of the present results and since RPF can now reliably be quantified along with routine haemograms, RPF might routinely be applied in the setting of cardiovascular risk prediction.

## Figures and Tables

**Table 1:** Baseline characteristics.

Clinical parameters	Overall (n=486)	MACE (n=52)	No MACE (n=434)	P-value
Age, years	64±13	68±13	64±13	0.04
Female, n(%)	154 (31.7)	18 (34.6)	136 (31.3)	0.631
Hypertension, n(%)	416 (85.6)	42 (80.8)	374 (86.4)	0.274
Diabetes mellitus, n(%)	140 (28.8)	19 (36.5)	121 (27.9)	0.196
Hypercholesterolaemia, n(%)	381 (78.4)	40 (76.9)	341 (78.8)	0.761
Active smoking, n(%)	140 (28.8)	12 (23.1)	128 (29.5)	0.334
Prior PCI, n(%)	116 (23.9)	16 (30.8)	100 (23.1)	0.220
Prior myocardial infarction, n(%)	127 (26.1)	20 (38.5)	107 (24.7)	0.033
PCI				
Due to STE-ACS, n(%)	124 (25.5)	12 (23.1)	112 (25.8)	0.563
Due to NSTE-ACS, n(%)	164 (33.7)	21 (40.4)	143 (32.9)	
Due to stable CAD, n(%)	198 (40.7)	19 (36.5)	179 (41.2)	
Cardiogenic shock, n(%)	10 (2.1)	5 (9.6)	5 (1.2)	P<0.001
DES, n(%)	315 (64.8)	27 (51.9)	288 (66.4)	0.039
Stents per patient	1 (1–2)	1 (1–2)	1 (1–2)	0.616
Total stent length, mm	24 (18–37.3)	24 (18–32)	24 (18–38)	0.985
Multivessel PCI, n(%)	55 (11.3)	5 (9.6)	50 (11.5)	0.682
TIMI composite bleeding, n(%)	39(8)	11 (21.2)	28 (6.5)	<0.001
Comedication				
Angiotensin converting enzyme-inhibitor, n(%)	234 (48.2)	21 (40.4)	213 (49.2)	0.230
Angiotensin receptor blocker, n(%)	93 (19.2)	8 (15.4)	85 (19.6)	0.462
(3-Blocker, n(%)	384 (79.2)	35 (67.3)	349 (80.6)	0.026
Calcium channel blocker, n(%)	50 (10.3)	5 (9.6)	45 (10.4)	0.862
Protone pump inhibitor, n(%)	402 (82.9)	43 (82.7)	359 (82.9)	0.969
Statin, n(%)	436 (89.7)	42 (80.8)	394(91)	0.021

Abbreviations: CAD: Coronary artery disease, PCI: Percutaneous coronary intervention, ST: Stent thrombosis, STE-ACS: ST-elevation-acute coronary syndrome, NSTE-ACS: Non ST-elevation-acute coronary syndrome, DES: Drug eluting stent.

**Table 2:** Blood testing.

Blood testing	Overall n=486	MACE n=52	No MACE n=434	P-value
Platelet count, Gi/l	229.2 ± 60.5	221.3 ± 57.7	230.1 ± 60.9	0.324
White blood cell count, Gi/l	9 (7.6–10.8)	9.9 (8–12.3)	8.8 (7.5–10.7)	0.011
Hemoglobin, g/dl	13.5 (12.2–14.5)	12.9 (11.5–14)	13.6 (12.2–14.6)	0.029
Glucose (at admission), mg/dl	116.5 (97.8–145.2)	126.4 (99–170)	116 (97.6–141.8)	0.120
C-reactive protein, mg/l	4.9 (1.9–13.8)	11.3 (2.9–32.4)	4.5 (1.8–11.7)	0.006
Creatinine, mg/dl	0.9 (0.8–1.1)	0.9 (0.8–1.3)	0.9 (0.8–1.1)	0.047
eGFR (MDRD)	70.1 (65.6–70.1)	70.1 (50.7–70.1)	70.1 (66.1–70.1)	0.011
Cholesterol, mg/dl	181.5 ± 46.6	171.1 ± 51.6	182.8 ± 45.9	0.106
Low-density lipoproteins, mg/dl	106 (80–134)	106.5 (76–136.5)	105 (80–133)	0.781
High-density lipoproteins, mg/dl	41.2 (35–52)	42 (36–47)	41 (35–52.7)	0.807
Troponin I, ng/ml	1–3 (0.1–13.4)	3.3 (0.2–25)	1.2 (0.1–12.5)	0.138
Decline in platelet count, Gi/l	5 (-8–24)	8 (-4–23)	5 (-8–24)	0.455
MPV, fl	10.7 (10.1–11.3)	10.8 (10.5–11.7)	10.6 (10.1–11.3)	0.017
RPF, %	3.4 (2.5–4.7)	4 (2.9–5.4)	3.3 (2.4–4.7)	0.013
MEA, U	31 (21–48)	30 (21.5–49.5)	31 (21–48)	0.972
VASP-P, PRI%	63.3 (48–74.93)	66.5 (53.5–78.5)	62.3 (47.2–74.8)	0.077

**Table 3:** Univariable logistic regression.

	MACE (OR (CI 95 %))	P-value	Bleeding (OR (CI 95 %))	P-value
Platelet function tests				
MEA	0.999 (0.980–1.017)	0.895	0.991 (0.970–1.013)	0.418
VASP-P	1.016 (1.000–1.032)	0.052	1.015 (0.997–1.034)	0.099
RPF	1.173 (1.040–1.324)	0.009	1.243 (1.091–1.415)	0.001
MPV	1.459 (1.059–2.008)	0.021	1.484 (1.035–2.127)	0.032
Clinical parameters				
Age	1.025 (1.001–1.049)	0.042	1.027 (1.000–1.055)	0.050
Gender	1.160 (0.633–2.127)	0.631	1.085 (0.542–2.174)	0.818
Body mass index	0.994 (0.934–1.058)	0.852	0.988 (0.921–1.061)	0.749
Bodyfat	0.987 (0.969–1.005)	0.147	0.996 (0.982–1.011)	0.633
Indication for PCI	1.205 (0.895–1.623)	0.219	1.190 (0.848–1.669)	0.315
Cardiogenic shock	9.128 (2.549–32.686)	0.001	32.38 (7.99–131.19)	<0.001
Hypertension	0.663 (0.315–1.392)	0.277	0.614 (0.270–1.398)	0.245
Diabetes mellitus	1.485 (0.813–2.711)	0.198	1.605 (0.815–3.160)	0.171
Hypercholesterolaemia	0.899 (0.453–1.784)	0.761	0.585 (0.285–1.198)	0.143
Active smoking	0.804 (0.561–1.152	0.235	0.920 (0.615–1.377)	0.686
History of PCI	0.674 (0.359–1.265)	0.219	2.925 (1.017–8.431)	0.046
History of myocardial infarction	1.904 (1.045–3.469)	0.035	0.782 (0.383–1.594)	0.498
History of atrial fibrillation	0.453 (0.197–1.041)	0.062	1.115 (0.328–3.791)	0.862
History of TIA/Stroke	1.539 (0.567–4.176)	0.398	1.156 (0.336–3.972)	0.818
Alcohol consumption	0.642 (0.412–1.000)	0.050	0.750 (0.462–1.220)	0.247
Heart rate at admission	1.010 (0.995–1.025)	0.205	1.021 (1.005–1.037)	0.011
Laboratory parameters				
Renal function (estimated GFR)	0.963 (0.942–0.984)	0.001	0.952 (0.930–0.974)	<0.001
Troponin I	1.007 (1.002–1.013)	0.009	1.008 (1.002–1.014)	0.006
Platelet count	0.998 (0.993–1.002)	0.323	0.995 (0.989–1.001)	0.088
Decline in platelet count	1.004 (0.996–1.012)	0.356	1.010 (1.001–1.019)	0.032
Haemoglobin	0.833 (0.704–0.987)	0.035	0.673 (0.555–0.816)	<0.001
Leucocyte count	1.093 (1.009–1.185)	0.029	1.255 (1.129–1.395)	<0.001
Glucose	1.004 (1.000–1.008)	0.061	1.009 (1.005–1.014)	<0.001
C-reactive protein	1.010 (1.004–1.016)	0.002	1.019 (1.012–1.025)	<0.001
Total cholesterol	0.995 (0.988–1.001)	0.104	0.995 (0.987–1.002)	0.163
LDL cholesterol	0.998 (0.990–1.006)	0.627	1.000 (0.991–1.009)	0.988
HDL cholesterol	0.992 (0.968–1.016)	0.492	0.979 (0.951–1.007)	0.143
Comedication				
Use of GPIIb/IIIa-blockers	1.079 (0.801–1.454)	0.617	1.871 (1.384–2.528)	<0.001
Type of Anticoagulant during PCI	1.234 (0.839–1.814)	0.285	0.749 (0.419–1.341)	0.330
ACE-inhibitors	1.429 (0.796.2.566)	0.232	1.738 (0.881–3.431)	0.111
Beta-blockers	0.501 (0.268–0.938)	0.031	0.438 (0.219–0.877)	0.020
Types of statins	0.706 (0.512–0.973)	0.033	0.966 (0.662–1.408)	0.856
PCI				
Vascular access site	1.415 (0.402–4.976)	0.589	-	-
Multivessel PCI	0.817 (0.310–2.151)	0.682	2.615 (1.169–5.849)	0.019
Number of stents used	0.977 (0.660–1.447)	0.909	1.355 (0.928–1.977)	0.116
Use of drug eluting stents	0.548 (0.307–0.977)	0.042	0.607 (0.314–1.174)	0.138
Total stent length	0.999 (0.982–1.016)	0.900	1.011 (0.994–1.027)	0.208
Composite in hospital bleeding	3.890 (1.805–8.384)	0.001	-	-

**Table 4:** **Ischaemic events during follow up according to a cut-off value of RPF = 3.35 %.** Comparison between the two groups with the use of the Chi^2^ test.

Ischaemic events	RPF<3.35 % (n=231)	RPF>3.35 % (n=255)	P-value
Death (cardiovascular), n (%)	7 (3.0)	11 (4.3)	ns
Myocardial infarction, n (%)	5 (2.2)	16 (6.3)	0.026
Revascularisation (unplanned), n (%)	6 (2.6)	21 (8.2)	0.007
Stent thromboses (definite and probable), n (%)	3 (1.3)	7 (2.7)	ns
Unstable angina, n (%)	3 (1.3)	3 (1.2)	ns
TIA/Stroke, n (%)	1 (0.4)	3 (1.2)	ns
Total, n (%)	25 (10.8)	61 (23.9)	<0.001
